# Supplementary material for: Topography of associations between cardiovascular risk factors and myelin loss in the ageing human brain
Source: Commun Biol. 2023 Apr 10;6:392. doi: 10.1038/s42003-023-04741-1 (PMC10086032; doi:10.1038/s42003-023-04741-1)
Supplement: Supplementary file 2 — Supplementary Information [file 42003_2023_4741_MOESM2_ESM.pdf]

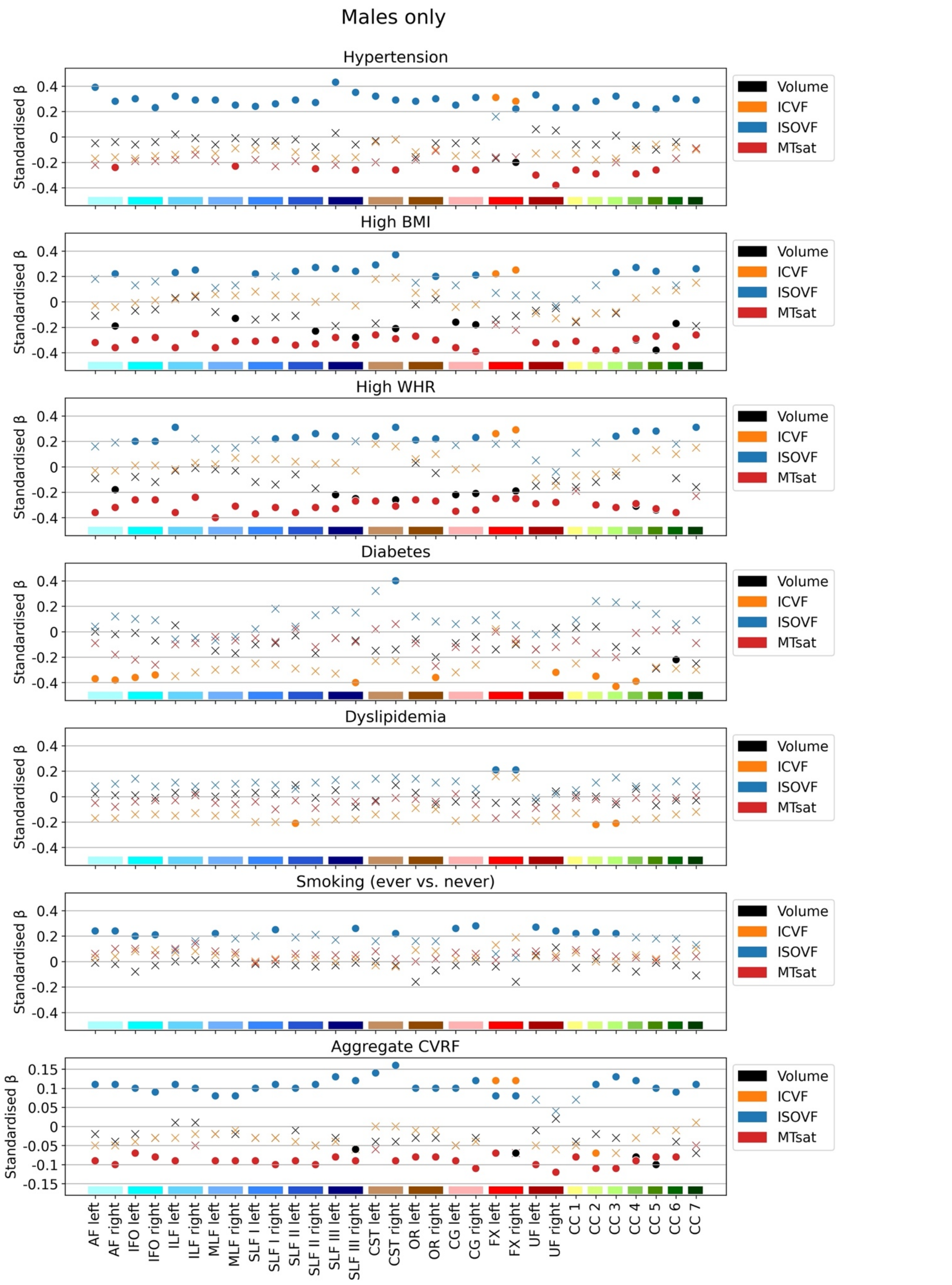

Supplementary Figure 1. White matter tract microstructure associations with cardiovascular risk factors in male participants only (n=543). Models were adjusted for age, age^2 and total intracranial volume. Standardised βs are shown as filled circles for significant associations (FDR-corrected p<0.05) and as crosses for non-significant associations (FDR-corrected p≥0.05). The x-axis contains the 31 tracts-of-interest coloured

and grouped, as in Fig. 1, by association tracts (shades of blue), projection tracts (brown), limbic tracts (pink/red), and corpus callosum (yellow/green). Abbreviations: AF = arcuate fasciculus, BMI = body mass index, CC = corpus callosum (1 = rostrum, 2 = genu, 3 = rostral body, 4 = anterior midbody, 5 = posterior midbody, 6 = isthmus, 7 = splenium), CG = cingulum bundle, CST = cortico-spinal tract, FX = fornix, ICVF = intra-cellular volume fraction, IFO = inferior fronto-occipital fasciculus, ILF = inferior longitudinal fasciculus, ISOVF = isotropic volume fraction, MLF = middle longitudinal fasciculus, MTsat = magnetization transfer saturation, OR = optic radiation, SLF = superior longitudinal fasciculus, UF = uncinate fasciculus, WHR = waist-to-hip ratio.

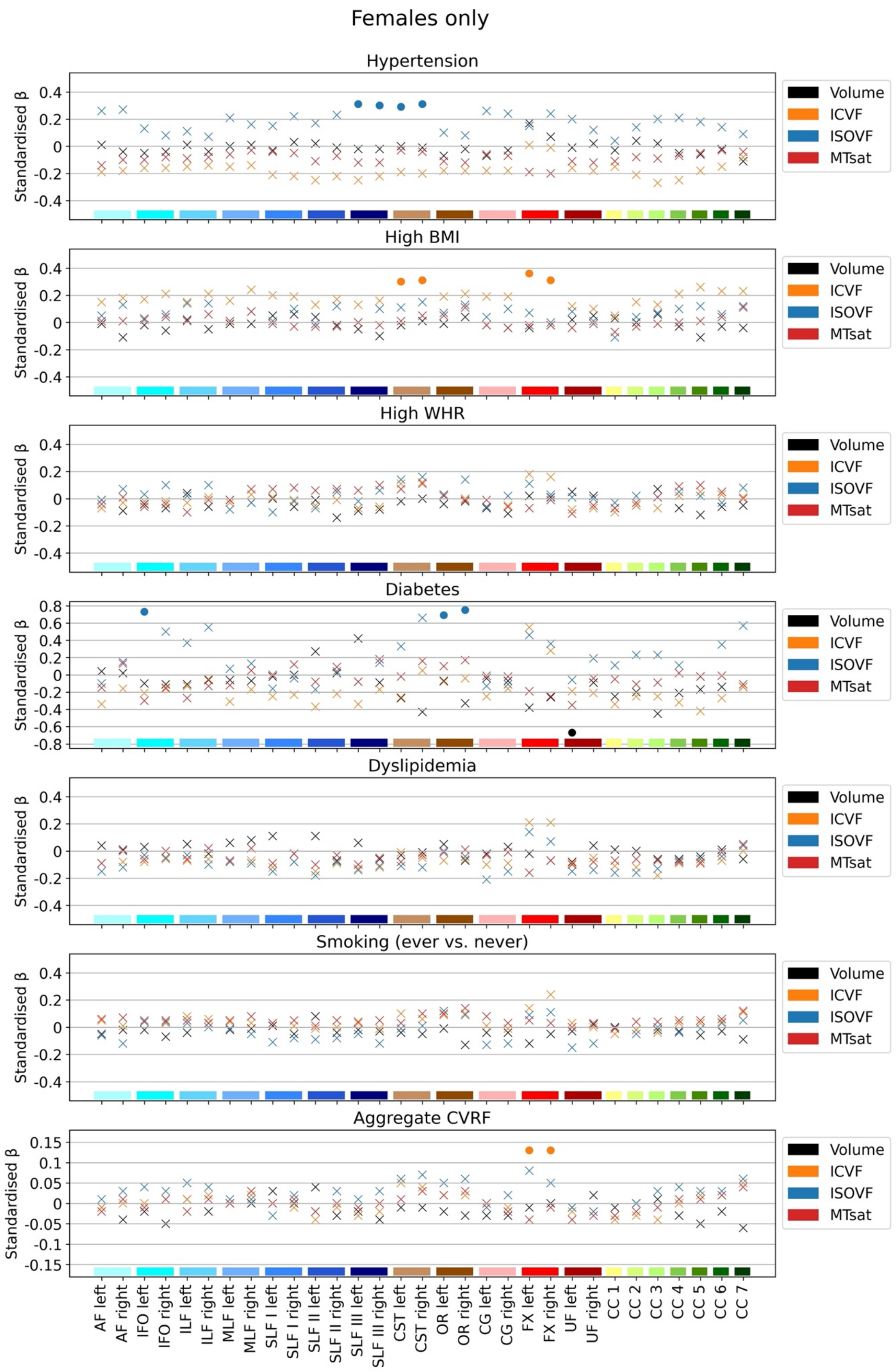

Supplementary Figure 2. White matter tract microstructure associations with cardiovascular risk factors in female participants only (n=561). Models were adjusted for age, age^2 and total intracranial volume. Standardised βs are shown as filled circles for significant associations (FDR-corrected $p<0.05$) and as crosses for non-significant associations (FDR-corrected $p\geq0.05$). The x-axis contains the 31 tracts-of-interest coloured

and grouped, as in Fig. 1, by association tracts (shades of blue), projection tracts (brown), limbic tracts (pink/red), and corpus callosum (yellow/green). Abbreviations: AF = arcuate fasciculus, BMI = body mass index, CC = corpus callosum (1 = rostrum, 2 = genu, 3 = rostral body, 4 = anterior midbody, 5 = posterior midbody, 6 = isthmus, 7 = splenium), CG = cingulum bundle, CST = cortico-spinal tract, FX = fornix, ICVF = intra-cellular volume fraction, IFO = inferior fronto-occipital fasciculus, ILF = inferior longitudinal fasciculus, ISOVF = isotropic volume fraction, MLF = middle longitudinal fasciculus, MTsat = magnetization transfer saturation, OR = optic radiation, SLF = superior longitudinal fasciculus, UF = uncinate fasciculus, WHR = waist-to-hip ratio.

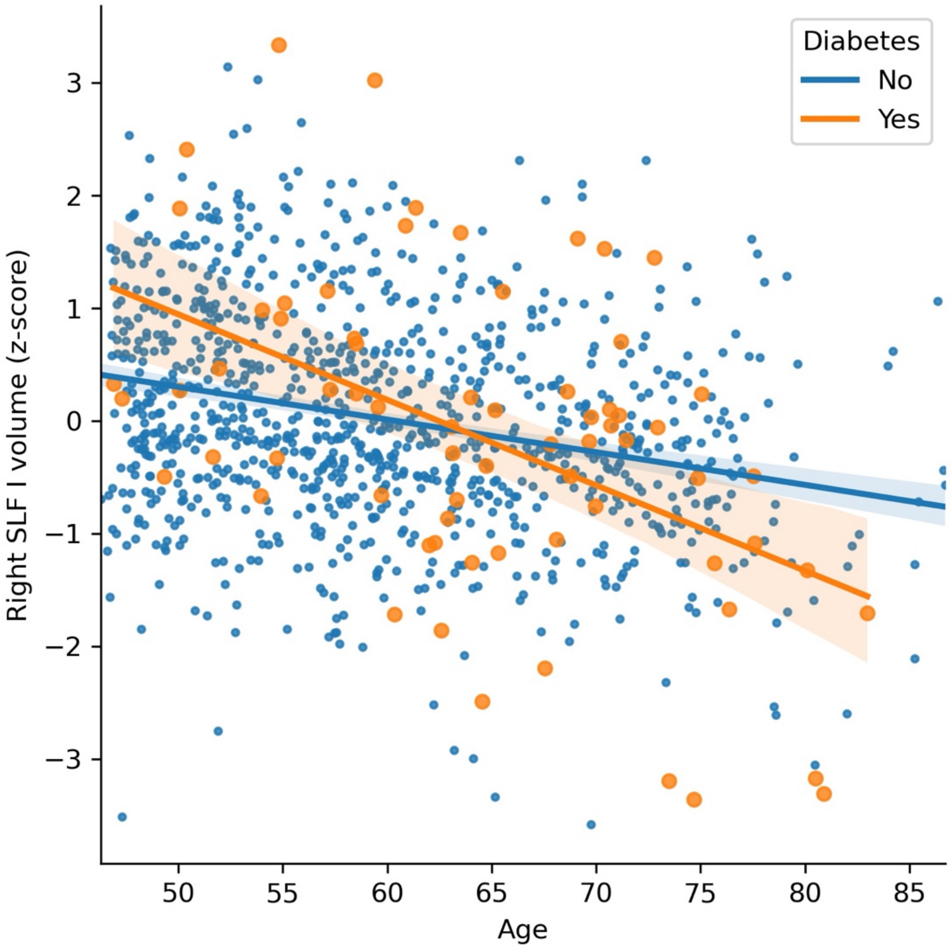

Supplementary Figure 3. To illustrate the age x diabetes interaction on tract volumes, right SLF I volume is plotted against age in participants with (orange) or without (blue) diabetes. The regression lines indicate model estimates with a 95% confidence interval. Abbreviations: SLF = superior longitudinal fasciculus.

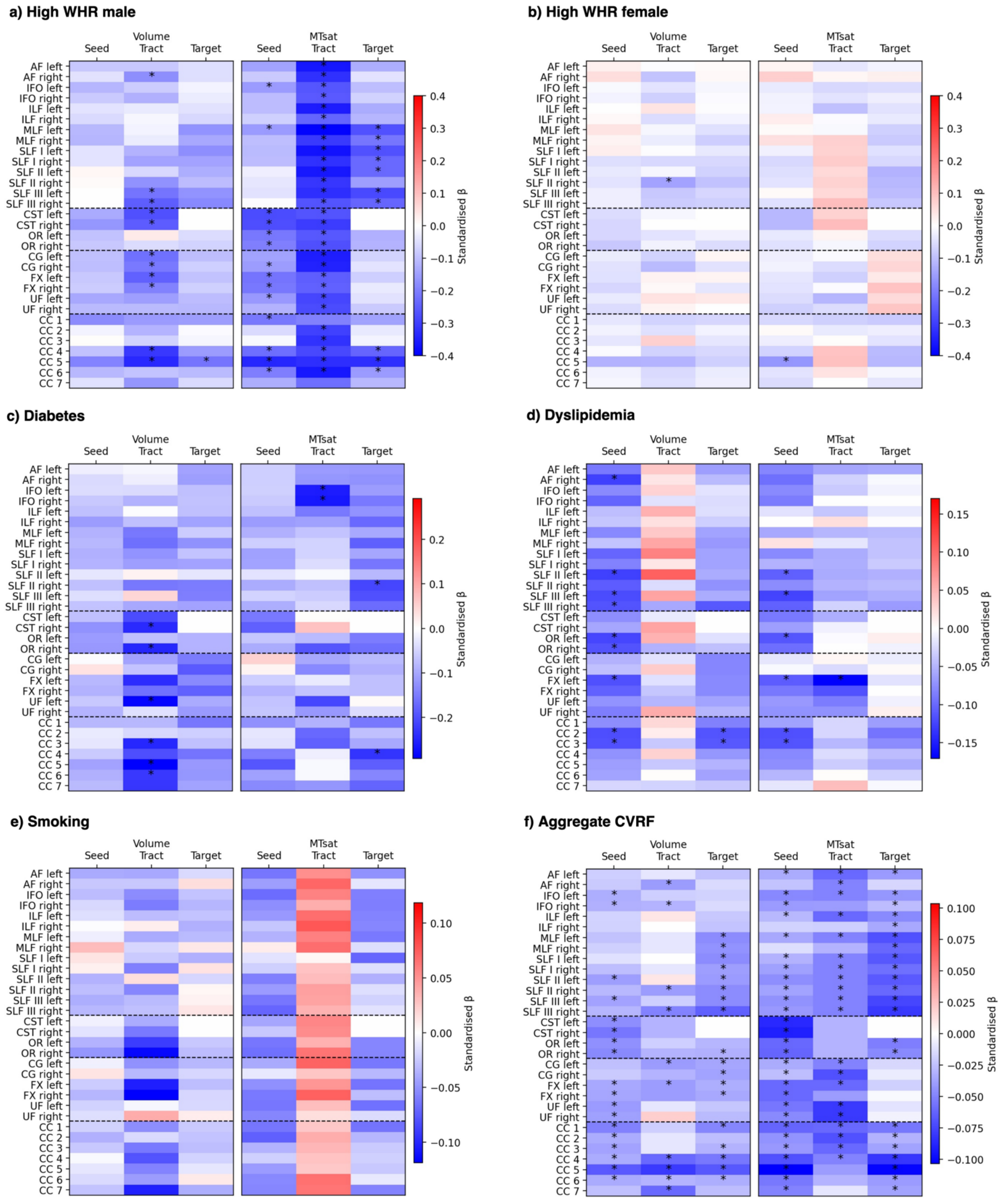

Supplementary Figure 4. CVRFs associations with the volume and MTsat in WM tracts and their GM seed and target regions (n=1104). Standardised βs are marked with an asterisk for significant associations (FDR-corrected p<0.05) GM and WM associations with high WHR in (**a**) male and (**b**) female participants, with (**c**) diabetes, (**d**) dyslipidemia, (**e**) smoking and (**f**) aCVRF in all participants. Abbreviations: A/PCG = anterior/posterior cingulate gyrus, AF = arcuate fasciculus, Amyg = amygdala, AnG = angular gyrus, A/L/M/POrG = anterior/lateral/middle/posterior orbital gyrus, Calc = calcarine cortex, CC = corpus callosum (1 = rostrum, 2 = genu, 3 = rostral body, 4 = anterior midbody, 5 = posterior midbody, 6 = isthmus,

7 = splenium), CG = cingulum bundle, CST = cortico-spinal tract, Ent. area = entorhinal area, FRP = frontal pole, FX = fornix, GM = grey matter, GRe = gyrus rectus, Hippoc = hippocampus, IFO = inferior fronto-occipital fasciculus, ILF = inferior longitudinal fasciculus, I/M/SOG = inferior/middle/superior occipital gyrus, I/M/S/TTG = inferior/middle/superior/transverse temporal gyrus, LiG = lingual gyrus, MFC = medial frontal cortex, M/SFG = middle/superior frontal gyrus, MLF = middle longitudinal fasciculus, (M)Po/PrG = post/precentral gyrus (medial segment), MTsat = magnetization transfer saturation, Op/Or/TrIFG = opercular/orbital/triangular part of inferior frontal gyrus, OCP = occipital pole, OFuG = occipital fusiform gyrus, OR = optic radiation, PHG = parahippocampal gyrus, PO = parietal operculum, Precun = precuneus, SCA = subcallosal area, SLF = superior longitudinal fasciculus, SMC = supplementary motor cortex, SMG = supramarginal gyrus, SPL = superior parietal lobule, TMP = temporal pole, UF = uncinate fasciculus, Ventral DC = ventral diencephalon, WHR = waist-to-hip ratio, WM = white matter.

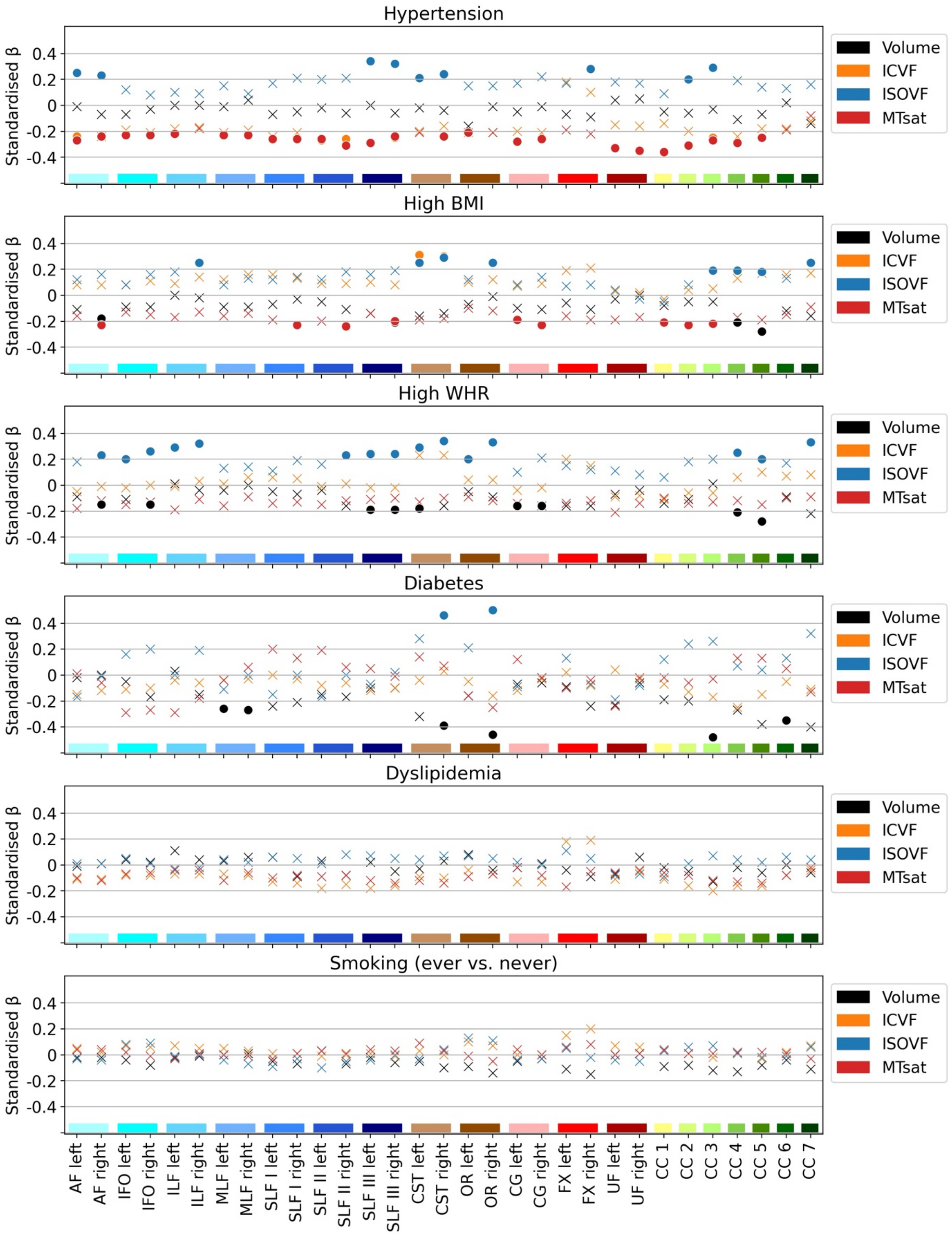

Supplementary Figure 5. **Control models of white matter tract microstructure associations with cardiovascular risk factors (n=600).** Models were adjusted for age, age^2, sex and total intracranial volume, only in participants with complete covariate data (same sample as Supplementary Figure 6). Standardised βs are shown as filled circles for significant associations (FDR-corrected p<0.05) and as crosses for non-significant associations (FDR-corrected p≥0.05). The x-axis contains the 31 tracts-of-interest coloured and grouped, as in Fig. 1, by association tracts (shades of blue), projection tracts (brown), limbic tracts (pink/red), and corpus callosum (yellow/green). Abbreviations: AF = arcuate fasciculus, BMI = body mass index, CC = corpus callosum (1 = rostrum, 2 = genu, 3 = rostral body, 4 = anterior midbody, 5 = posterior midbody,

6 = isthmus, 7 = splenium), CG = cingulum bundle, CST = cortico-spinal tract, FX = fornix, ICVF = intra-cellular volume fraction, IFO = inferior fronto-occipital fasciculus, ILF = inferior longitudinal fasciculus, ISOVF = isotropic volume fraction, MLF = middle longitudinal fasciculus, MTsat = magnetization transfer saturation, OR = optic radiation, SLF = superior longitudinal fasciculus, UF = uncinate fasciculus, WHR = waist-to-hip ratio.

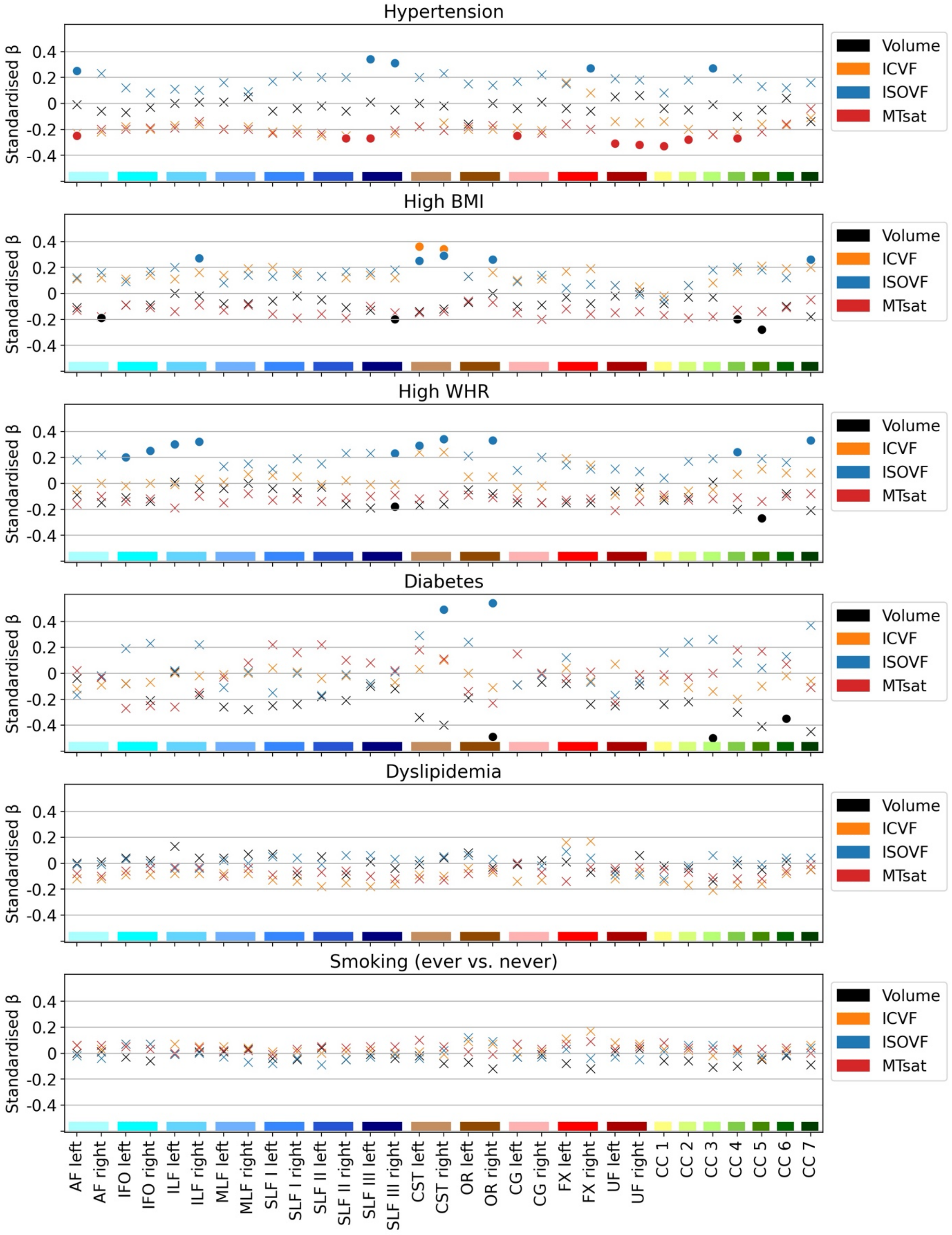

Supplementary Figure 6. **Fully adjusted models of white matter tract microstructure associations with cardiovascular risk factors (n=600)**. Models were adjusted for age, age^2, sex, total intracranial volume, educational level, ApoE risk, recent major depressive disorder with atypical or melancholic episodes, self-reported alcohol consumption and measured physical activity. Standardised βs are shown as filled circles for significant associations (FDR-corrected p<0.05) and as crosses for non-significant associations (FDR-corrected p≥0.05). The x-axis contains the 31 tracts-of-interest coloured and grouped, as in Fig. 1, by association tracts (shades of blue), projection tracts (brown), limbic tracts (pink/red), and corpus callosum (yellow/green). Abbreviations: AF = arcuate fasciculus, BMI = body mass index, CC = corpus callosum (1 = rostrum, 2 = genu, 3 = rostral body, 4 = anterior midbody, 5 = posterior midbody, 6 = isthmus, 7 = splenium), CG = cingulum bundle, CST = cortico-spinal tract, FX = fornix, ICVF = intra-cellular volume fraction, IFO = inferior fronto-occipital fasciculus, ILF = inferior longitudinal fasciculus, ISOVF = isotropic volume fraction, MLF = middle longitudinal fasciculus, MTsat = magnetization transfer saturation, OR = optic radiation, SLF = superior longitudinal fasciculus, UF = uncinate fasciculus, WHR = waist-to-hip ratio.

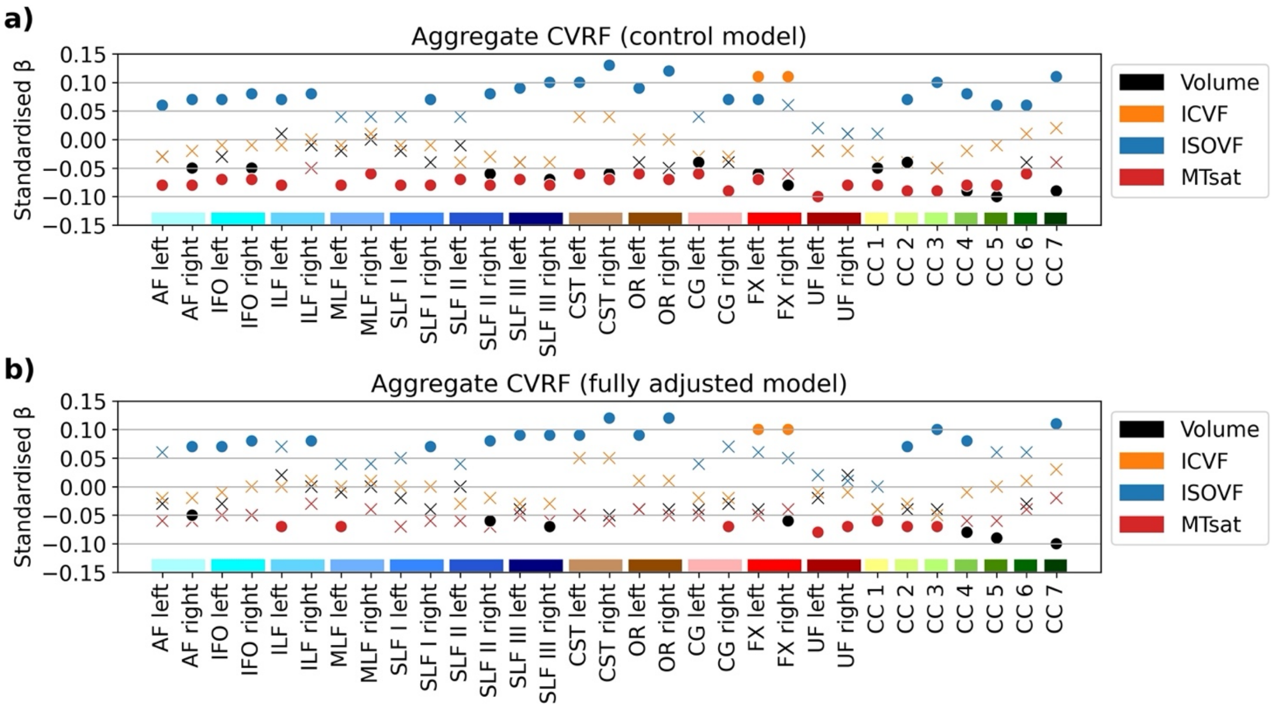

Supplementary Figure 7. **White matter tract microstructure associations with aggregate cardiovascular risk factor score in control and fully adjusted models.** Models were adjusted for **a)** age, age^2, sex and total intracranial volume, and **b)** additionally for educational level, ApoE risk, recent major depressive disorder with atypical or melancholic episodes, self-reported alcohol consumption and measured physical activity. Both **a)** and **b)** analyses included only participants with complete covariate data (n=600). Standardised βs are shown as filled circles for significant associations (FDR-corrected p<0.05) and as crosses for non-significant associations (FDR-corrected p≥0.05). The x-axis contains the 31 tracts-of-interest coloured and grouped, as in Fig. 1, by association tracts (shades of blue), projection tracts (brown), limbic tracts (pink/red), and corpus callosum (yellow/green). Abbreviations: AF = arcuate fasciculus, BMI = body mass index, CC = corpus callosum (1 = rostrum, 2 = genu, 3 = rostral body, 4 = anterior midbody, 5 = posterior midbody, 6 = isthmus, 7 = splenium), CG = cingulum bundle, CST = cortico-spinal tract, FX = fornix, ICVF = intra-cellular volume fraction, IFO = inferior fronto-occipital fasciculus, ILF = inferior longitudinal fasciculus, ISOVF = isotropic volume fraction, MLF = middle longitudinal fasciculus, MTsat = magnetization transfer saturation, OR = optic radiation, SLF = superior longitudinal fasciculus, UF = uncinate fasciculus, WHR = waist-to-hip ratio.

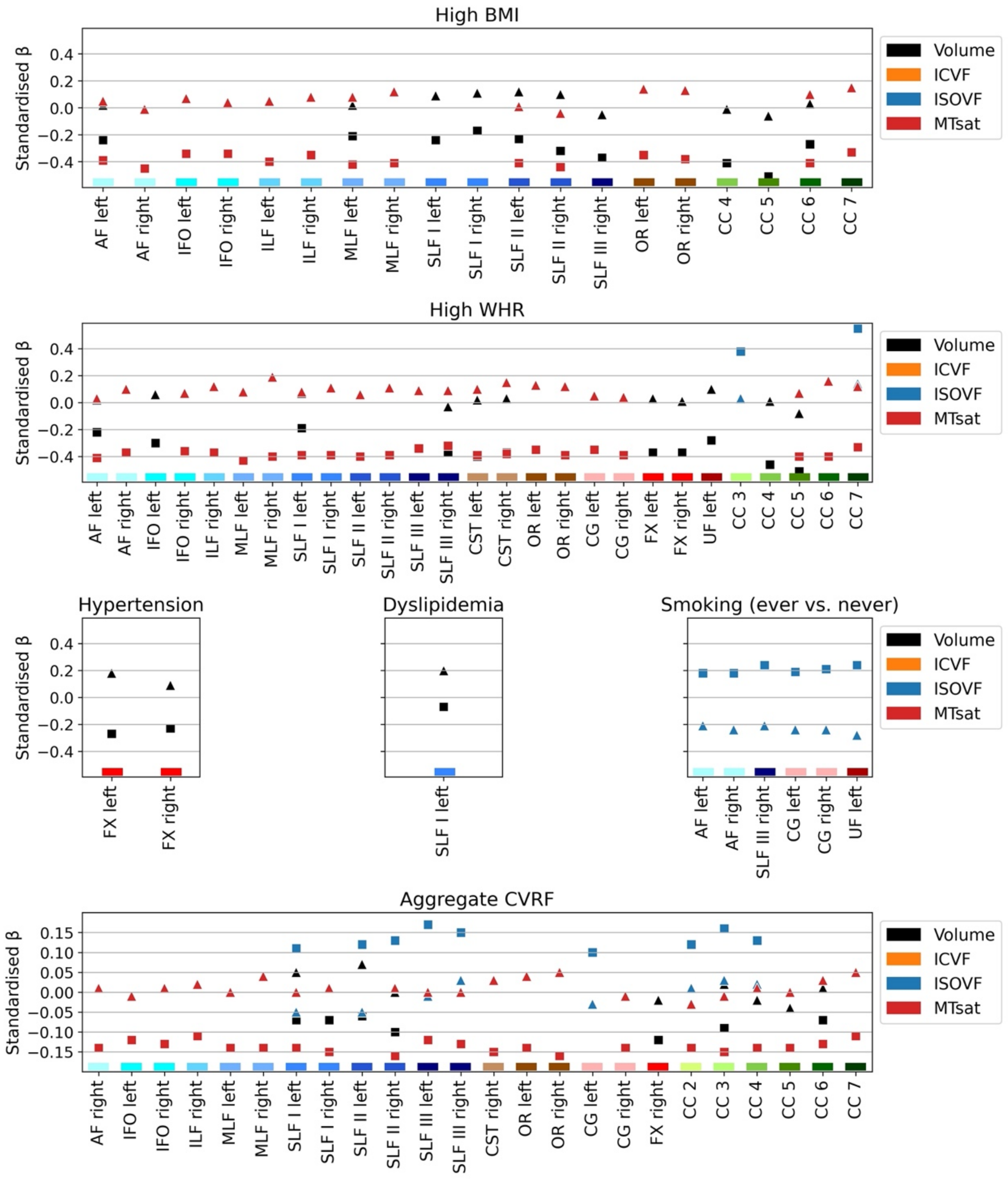

Supplementary Figure 8. Control models where the CVRF x sex interaction was significant (FDR-corrected p<0.05). Standardised βs of the association between white matter microstructure and CVRF are shown for male (squares) and female (triangles) participants separately. All models were performed only in participants with complete covariate data (n=600) and included the main effects of sex and CVRF, as well as the interaction term, and age, age^2 and total intracranial volume as covariates. The x-axis contains the 31 tracts-of-interest coloured and grouped, as in Fig. 1, by association tracts (shades of blue), projection tracts (brown), limbic tracts (pink/red), and corpus callosum (yellow/green). Abbreviations: AF = arcuate fasciculus, BMI = body mass index, CC = corpus callosum (1 = rostrum, 2 = genu, 3 = rostral body, 4 = anterior midbody, 5 = posterior midbody, 6 = isthmus, 7 = splenium), CG = cingulum bundle, CST = cortico-spinal tract, FX = fornix, ICVF = intra-cellular volume fraction, IFO = inferior fronto-occipital fasciculus, ILF = inferior longitudinal fasciculus, ISOVF = isotropic volume fraction, MLF = middle longitudinal fasciculus, MTsat = magnetization transfer saturation, OR = optic radiation, SLF = superior longitudinal fasciculus, UF = uncinate fasciculus, WHR = waist-to-hip ratio.

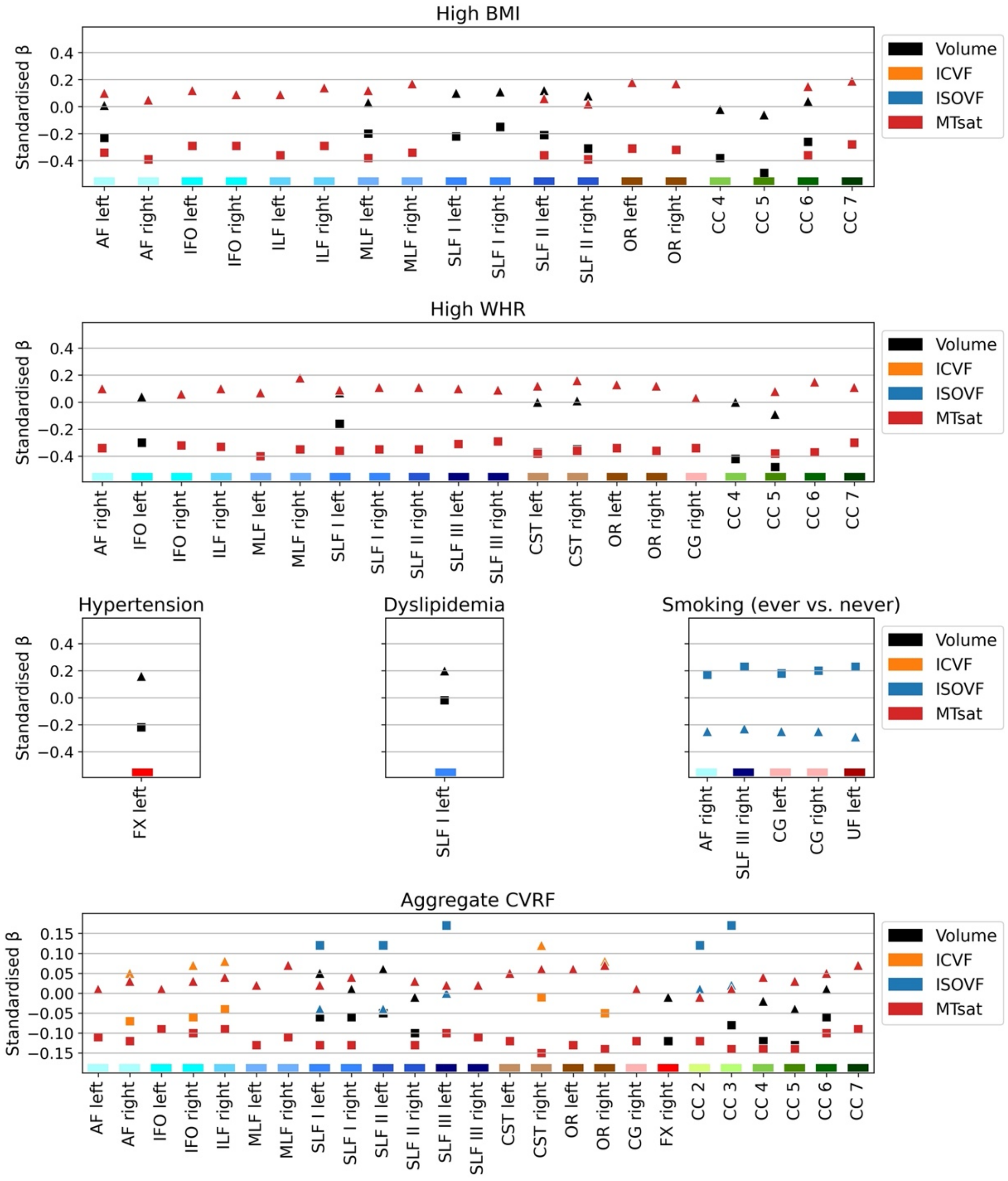

Supplementary Figure 9. Fully adjusted models where the CVRF x sex interaction was significant (FDR-corrected p<0.05). Standardised βs of the association between white matter microstructure and CVRF are shown for male (squares) and female (triangles) participants separately (total n=600). All models included the main effects of sex and CVRF, as well as the interaction term, and age, age^2, total intracranial volume, educational level, ApoE risk, recent major depressive disorder with atypical or melancholic episodes, self-reported alcohol consumption and measured physical activity as covariates. The x-axis contains the 31 tracts-of-interest coloured and grouped, as in Fig. 1, by association tracts (shades of blue), projection tracts (brown), limbic tracts (pink/red), and corpus callosum (yellow/green). Abbreviations: AF = arcuate fasciculus, BMI = body mass index, CC = corpus callosum (1 = rostrum, 2 = genu, 3 = rostral body, 4 = anterior midbody, 5 = posterior midbody, 6 = isthmus, 7 = splenium), CG = cingulum bundle, CST = cortico-spinal tract, FX = fornix, ICVF = intra-cellular volume fraction, IFO = inferior fronto-occipital fasciculus, ILF = inferior longitudinal fasciculus, ISOVF = isotropic volume fraction, MLF = middle longitudinal fasciculus, MTsat = magnetization transfer saturation, OR = optic radiation, SLF = superior longitudinal fasciculus, UF = uncinate fasciculus, WHR = waist-to-hip ratio.

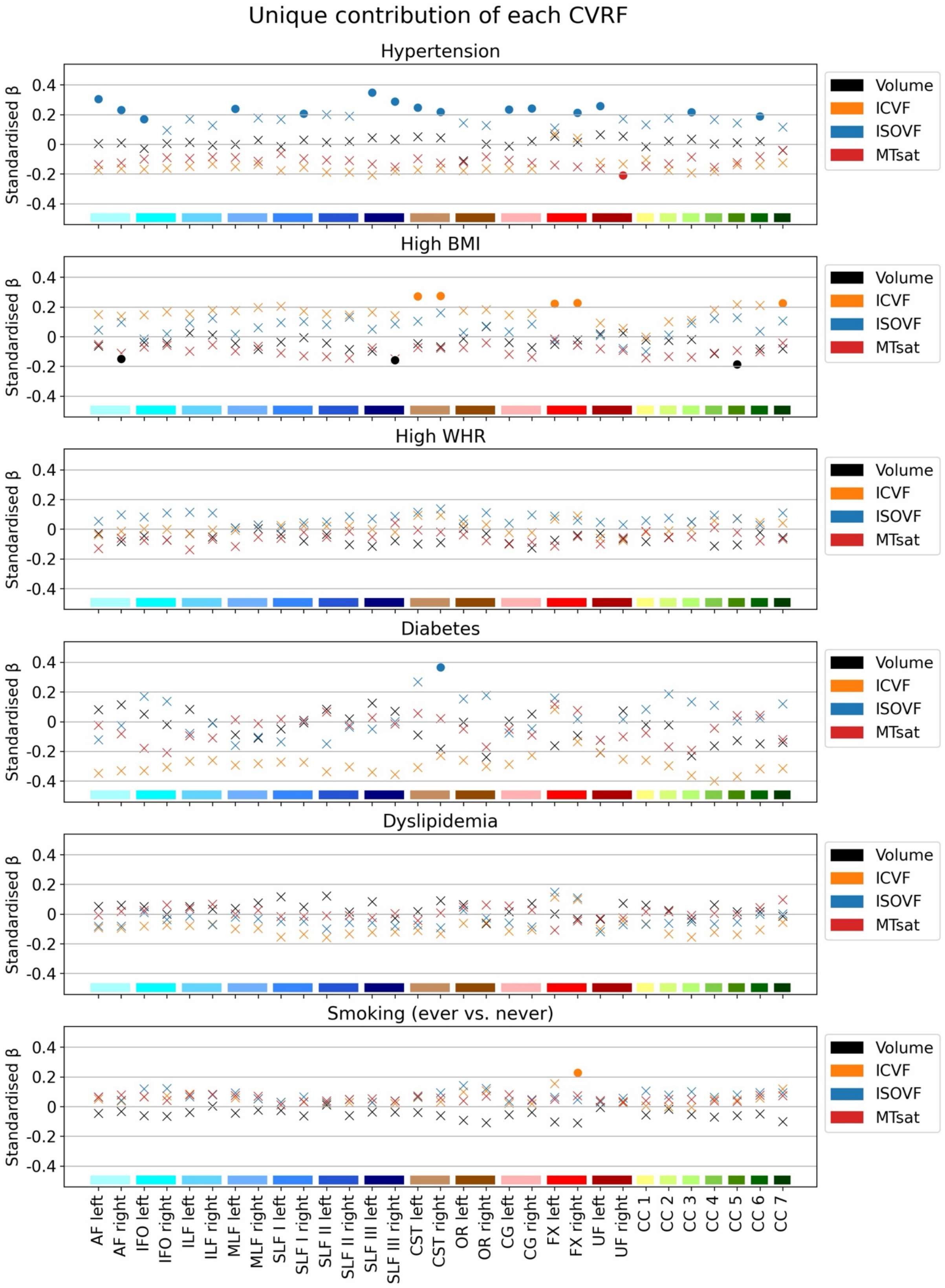

Supplementary Figure 10. Unique contribution of each cardiovascular risk factor to white matter tract microstructure variance, adjusting for the other five risk factors (n=1034). Models included age, age^2, sex, total intracranial volume, hypertension, high BMI, high WHR, diabetes, dyslipidemia, and smoking as regressors. Standardised βs associated with each factor are shown as filled circles for significant associations (FDR-corrected $p<0.05$) and as crosses for non-significant associations (FDR-corrected $p\geq0.05$). The x-axis contains the 31 tracts-of-interest coloured and grouped, as in Fig. 1, by association tracts (shades of blue),

projection tracts (brown), limbic tracts (pink/red), and corpus callosum (yellow/green). Abbreviations: AF = arcuate fasciculus, BMI = body mass index, CC = corpus callosum (1 = rostrum, 2 = genu, 3 = rostral body, 4 = anterior midbody, 5 = posterior midbody, 6 = isthmus, 7 = splenium), CG = cingulum bundle, CST = cortico-spinal tract, CVRF = cardiovascular risk factor, FX = fornix, ICVF = intra-cellular volume fraction, IFO = inferior fronto-occipital fasciculus, ILF = inferior longitudinal fasciculus, ISOVF = isotropic volume fraction, MLF = middle longitudinal fasciculus, MTsat = magnetization transfer saturation, OR = optic radiation, SLF = superior longitudinal fasciculus, UF = uncinate fasciculus, WHR = waist-to-hip ratio.
