# Supplementary material for: Topography of associations between cardiovascular risk factors and myelin loss in the ageing human brain
Source: Commun Biol. 2023 Apr 10;6:392. doi: 10.1038/s42003-023-04741-1 (PMC10086032; doi:10.1038/s42003-023-04741-1)
Supplement: Supplementary file 3 — Description of Additional Supplementary Files [file 42003_2023_4741_MOESM3_ESM.pdf]

Description of Additional Supplementary Files

File name: Supplementary Data 1
Description: For each magnetic resonance imaging map and white matter tract, sample size after outlier removal, mean value and standard deviation across the sample.

File name: Supplementary Data 2
Description: Standardised β and p-value estimates for linear and quadratic effects of age on white matter microstructure.

File name: Supplementary Data 3
Description: Standardised β and p-value estimates for white matter microstructure associations with cardiovascular risk factors, their interaction with sex and age, and their unique contribution (i.e. in models adjusted for the other five CVRFs).

File name: Supplementary Data 4
Description: Standardised β and p-value estimates for white matter microstructure associations with aggregate cardiovascular risk score and its interaction with sex and age.

File name: Supplementary Data 5
Description: Standardised βs and p-values associated with age, age^2, diabetes, and their interaction terms in linear regression models of white matter tracts volume, shown only in models where interactions were significant.

File name: Supplementary Data 6
Description: Sample size, standardised β and p-value estimates for white matter microstructure associations with education level, ApoE genetic risk, atypical and melancholic major depressive disorder subtypes, moderate-to-vigorous physical activity amount, alcohol consumption, cardiovascular risk factors and their interaction with sex and age.

File name: Supplementary Data 7
Description: Control models standardised β and p-value estimated only from individuals with complete covariate data, along with sample size and number of missing data in each covariate.

File name: Supplementary Data 8
Description: Standardised β and p-value estimates for interaction between covariates and cardiovascular risk factors.

File name: Supplementary Data 9
Description: Sample sizes, standardised β and p-value estimates for associations between white matter myelin (MTsat) and lifestyle factors.
