# Supplementary material for: Topography of associations between cardiovascular risk factors and myelin loss in the ageing human brain
Source: Commun Biol. 2023 Apr 10;6:392. doi: 10.1038/s42003-023-04741-1 (PMC10086032; doi:10.1038/s42003-023-04741-1)
Supplement: Supplementary file 5 — Reporting Summary [file 42003_2023_4741_MOESM5_ESM.pdf]

nature portfolio

Corresponding author(s): Bogdan Draganski

Last updated by author(s): Mar 8, 2023

Reporting Summary

Nature Portfolio wishes to improve the reproducibility of the work that we publish. This form provides structure for consistency and transparency in reporting. For further information on Nature Portfolio policies, see our Editorial Policies and the Editorial Policy Checklist.

Statistics

For all statistical analyses, confirm that the following items are present in the figure legend, table legend, main text, or Methods section.

n/a	Confirmed	
☐	☒	The exact sample size (*n*) for each experimental group/condition, given as a discrete number and unit of measurement
☒	☐	A statement on whether measurements were taken from distinct samples or whether the same sample was measured repeatedly
☐	☒	The statistical test(s) used AND whether they are one- or two-sided<br>*Only common tests should be described solely by name; describe more complex techniques in the Methods section.*
☐	☒	A description of all covariates tested
☐	☒	A description of any assumptions or corrections, such as tests of normality and adjustment for multiple comparisons
☐	☒	A full description of the statistical parameters including central tendency (e.g. means) or other basic estimates (e.g. regression coefficient) AND variation (e.g. standard deviation) or associated estimates of uncertainty (e.g. confidence intervals)
☐	☒	For null hypothesis testing, the test statistic (e.g. *F*, *t*, *r*) with confidence intervals, effect sizes, degrees of freedom and *P* value noted<br>*Give P values as exact values whenever suitable.*
☒	☐	For Bayesian analysis, information on the choice of priors and Markov chain Monte Carlo settings
☒	☐	For hierarchical and complex designs, identification of the appropriate level for tests and full reporting of outcomes
☐	☒	Estimates of effect sizes (e.g. Cohen's *d*, Pearson's *r*), indicating how they were calculated

Our web collection on statistics for biologists contains articles on many of the points above.

Software and code

Policy information about availability of computer code

Data collection	No software was used
Data analysis	Python v3.8.8, pandas v1.2.4, numpy v1.20.1, statsmodels v0.12.2, scipy v1.6.2

For manuscripts utilizing custom algorithms or software that are central to the research but not yet described in published literature, software must be made available to editors and reviewers. We strongly encourage code deposition in a community repository (e.g. GitHub). See the Nature Portfolio guidelines for submitting code & software for further information.

Data

Policy information about availability of data

All manuscripts must include a data availability statement. This statement should provide the following information, where applicable:

- Accession codes, unique identifiers, or web links for publicly available datasets
- A description of any restrictions on data availability
- For clinical datasets or third party data, please ensure that the statement adheres to our policy

The CoLaus|PsyCoLaus cohort data used in this study cannot be fully shared as they contain potentially sensitive patient information. As discussed with the competent authority, the Research Ethic Committee of the Canton of Vaud, transferring or directly sharing this data would be a violation of the Swiss legislation aiming to protect the personal rights of participants. Non-identifiable, individual-level data are available for interested researchers, who meet the criteria for access

to confidential data sharing, from the CoLaus Datacenter (CHUV, Lausanne, Switzerland). Instructions for gaining access to the CoLaus data used in this study are available at https://www.colaus-psycolaus.ch/professionals/how-to-collaborate/.

Human research participants

Policy information about studies involving human research participants and Sex and Gender in Research.

Reporting on sex and gender	Biological sex was collected and sex-specific analyses were performed as described in details in the manuscript.
Population characteristics	Covariate relevant information is described in the Methods and summarised in Table 1. Mean age was 60.1 (standard deviation 9.1)
Recruitment	6734 individuals aged 35 to 75 years were recruited between 2003 and 2006 (baseline) from the civil registry of the city of Lausanne in Switzerland. There were three follow-up evaluations, one from 2009 to 2013 (first follow-up), a second one from 2014 to 2018 (second follow-up) and another one from 2018 to 2022 (third follow-up). During the second follow-up, 1324 participants also took part in the brain magnetic resonance imaging (MRI) investigation (BrainLaus study), among whom 1167 participants completed the full MRI acquisition protocol and 157 interrupted it before the end of the protocol.
Ethics oversight	The institutional Ethics Committee of the University of Lausanne, which afterwards became the Ethics Commission of Canton Vaud (www.cer-vd.ch)

Note that full information on the approval of the study protocol must also be provided in the manuscript.

Field-specific reporting

Please select the one below that is the best fit for your research. If you are not sure, read the appropriate sections before making your selection.

☒ Life sciences ☐ Behavioural & social sciences ☐ Ecological, evolutionary & environmental sciences

For a reference copy of the document with all sections, see nature.com/documents/nr-reporting-summary-flat.pdf

Life sciences study design

All studies must disclose on these points even when the disclosure is negative.

Sample size	The baseline sample of 6734 individuals was determined to be representative of the Lausanne population (ca. 120,000 inhabitants in 2003). The brain MRI sub-sample of 1324 individuals was based on maximal use of the MRI facility during the follow-up period (2014-2018) to maximise sample size
Data exclusions	157 participants interrupted MRI acquisition before the end of the protocol. On the remaining images, we performed a quantitative analysis of image degradation due to head motion using the quality index introduced in (Lutti et al., 2022) and a visual inspection for gross abnormalities. The 63 participants' data that did not meet the criteria outlined in (Trofimova et al, 2021) were excluded from subsequent analysis.
Replication	No replication was attempted in independent datasets.
Randomization	There were no experimental groups in our study. This is a cross-sectional association study in a population-based cohort.
Blinding	Blinding was not relevant since there were no experimental groups in our study.

Reporting for specific materials, systems and methods

We require information from authors about some types of materials, experimental systems and methods used in many studies. Here, indicate whether each material, system or method listed is relevant to your study. If you are not sure if a list item applies to your research, read the appropriate section before selecting a response.

Materials & experimental systems

n/a	Involved in the study
☒	☐ Antibodies
☒	☐ Eukaryotic cell lines
☒	☐ Palaeontology and archaeology
☒	☐ Animals and other organisms
☒	☐ Clinical data
☒	☐ Dual use research of concern

Methods

n/a	Involved in the study
☒	☐ ChIP-seq
☒	☐ Flow cytometry
☐	☒ MRI-based neuroimaging

Magnetic resonance imaging

Experimental design

Design type: Structural MRI

Design specifications: Not relevant (no trials in the study)

Behavioral performance measures: Not relevant (no trials in the study)

Acquisition

Imaging type(s): Structural

Field strength: 3T

Sequence & imaging parameters: The quantitative MRI protocol included three multi-echo 3D fast low angle shot (FLASH) acquisitions with magnetization transfer-weighted (MTw: TR = 24.5 ms, α = 6°), proton density-weighted (PDw: TR = 24.5 ms, α = 6°) and T1-weighted (T1w: TR = 24.5 ms, α = 21°) contrasts with 1 mm isotropic resolution. We used B1 maps computed with the 3D echo-planar spin-echo and stimulated echo images (4 mm resolution, TE = 39.06 ms, TR = 500 ms) to correct for the effects of RF transmit field inhomogeneities.
The diffusion-weighted imaging (DWI) protocol consisted of a 2D echo-planar sequence with the following parameters: TR=7400 ms, TE = 69ms, parallel GRAPPA acceleration factor = 2, FoV=192×212mm2, voxel size = 2 × 2 × 2 mm, matrix size=96×106, 70 axial slices, 118 gradient directions (15 at b = 650 s/mm2, 30 at b = 1000 s/mm2, 60 at b= 2000 s/mm2 and 13 at b = 0 interleaved throughout the acquisition). We also acquired B0-field maps (2D double-echo FLASH sequence with slice thickness = 2 mm, TR = 1020 ms, TE1/TE2 = 10/12.46 ms, α = 90°, BW = 260 Hz/pixel) to correct for geometric distortions in EPI data.

Area of acquisition: Whole brain scan

Diffusion MRI: ☒ Used ☐ Not used

Parameters: The diffusion-weighted imaging (DWI) protocol consisted of a 2D echo-planar sequence with the following parameters: TR=7400 ms, TE = 69ms, parallel GRAPPA acceleration factor = 2, FoV=192×212mm2, voxel size = 2 × 2 × 2 mm, matrix size=96×106, 70 axial slices, 118 gradient directions (15 at b = 650 s/mm2, 30 at b = 1000 s/mm2, 60 at b= 2000 s/mm2 and 13 at b = 0 interleaved throughout the acquisition).

Preprocessing

Preprocessing software: Quantitative MRI data were processed in the framework of Statistical Parametric Mapping SPM12 (www.fil.ion.ucl.ac.uk/spm; Wellcome Trust Centre for Neuroimaging, London) using customized MATLAB tools (The Mathworks, Sherborn, MA, USA). DWI data were preprocessed with MRtrix3 v3.3.7 and FSL 5.0.

Normalization: Quantitative MRI: SPM12's diffeomorphic "geodesic shoot" registration (Ashburner and Friston, 2011). Alignment of DWI to the magnetization transfer images: SPM12's rigid body registration.

Normalization template: Subject-specific template ("shoot" template in SPM12)

Noise and artifact removal: We performed a quantitative analysis of image degradation due to head motion using the quality index introduced in (Lutti et al., 2022) and a visual inspection for gross abnormalities. The participants' data that did not meet the criteria outlined in (Trofimova et al., 2021) were excluded from subsequent analysis.
DWI data were preprocessed with MRtrix3 including denoising and Gibbs ringing artefacts removal. We corrected for eddy current distortions and subject movements with the FSL 5.0 EDDY tool. For EPI susceptibility distortion correction, we used the acquired B0 maps with the SPM FieldMap toolbox. Bias field was estimated from the mean b = 0 images and corrected for in all DWI data.

Volume censoring: Not relevant

Statistical modeling & inference

Model type and settings	Multivariate linear regression, hierarchical clustering
Effect(s) tested	Associations between brain indices and cardiovascular risk factors as standardised betas (effect sizes)

Specify type of analysis: ☐ Whole brain ☒ ROI-based ☐ Both

Anatomical location(s)	White matter tracts segmented with TractSeg
Statistic type for inference (See Eklund et al. 2016)	Tract-wise average values analysed
Correction	False discovery rate (Benjamini & Hochberg, 1995)

Models & analysis

n/a	Involved in the study
☒	☐ Functional and/or effective connectivity
☒	☐ Graph analysis
☐	☒ Multivariate modeling or predictive analysis

Multivariate modeling and predictive analysis	Independent variables were tract-average MRI indices
